# International Conference on Emerging Infectious Diseases 2015 Poster and Oral Presentation Abstracts

**DOI:** 10.3201/eid2109.NN2109

**Published:** 2015-09

**Keywords:** iceid, conference, emerging infections, abstracts

Emerging Infectious Diseases is providing access to these abstracts on behalf of the ICEID 2015 program committee (http://www.iceid.org), which performed peer review. Members of the 2015 ICEID Scientific Committee and a list of peer reviewers are provided at the end of the PDF document.

Emerging Infectious Diseases has not edited or proofread these materials and is not responsible for inaccuracies or omissions. All information is subject to change. Comments and corrections should be brought to the attention of the authors.

